# Subchronic Exposure to Microcystin-LR Induces Hepatic Inflammation, Oxidative Stress, and Lipid Metabolic Disorders in Darkbarbel Catfish (*Tachysurus vachelli*)

**DOI:** 10.3390/toxins17060300

**Published:** 2025-06-12

**Authors:** Huaxing Zhou, Tong Li, Huan Wang, Ye Zhang, Yuting Hu, Amei Liu, Guoqing Duan

**Affiliations:** Anhui Key Laboratory of Aquaculture and Stock Enhancement, Fisheries Research Institution, Anhui Academy of Agricultural Sciences, Hefei 230031, China; litong@aaas.org.cn (T.L.); wanghuan@aaas.org.cn (H.W.); zhangye@aaas.org.cn (Y.Z.); huyuting@aaas.org.cn (Y.H.); liuamei@aaas.org.cn (A.L.)

**Keywords:** microcystin-LR, subchronic exposure, environmentally relevant concentration, hepatic inflammation, oxidative stress, lipid metabolic disorders

## Abstract

Microcystin-leucine arginine (MC-LR) is a prominent water pollutant known for its potent hepatic toxicity. However, the effects of subchronic exposure to environmentally relevant concentrations of MC-LR on the fish liver remain poorly understood. This study aimed to systematically evaluate the impact of subchronic MC-LR exposure on the liver of darkbarbel catfish (*Tachysurus vachelli*). A total of 270 one-year-old fish were exposed to MC-LR (0, 2, and 5 μg/L) for 28 days and sampled on days 14 (D14) and 28 (D28). Histopathological analysis revealed marked hepatic inflammation in the MC-LR treatment groups, manifested as cellular degeneration, hyperemia, and inflammation. MC-LR exposure induced oxidative stress, evidenced by elevated malondialdehyde (MDA) levels and compensatory upregulation of superoxide dismutase (SOD) activity on D28. While hepatic lipid profiles were not altered by low-dose MC-LR, significant elevation of low-density lipoprotein cholesterol (LDL-C) specifically on D28 indicated incipient lipid metabolic disorder. Metabolomic analysis demonstrated a higher sensitivity, highlighting the stress response of the liver to low-dose MC-LR exposure. The results suggest MC-LR exposure disrupted hepatic phosphatidylcholine (PC) biosynthesis and inhibited lipoprotein formation, thereby impairing lipid transport and contributing to lipid metabolic disorders. In summary, subchronic exposure to environmentally relevant concentrations of MC-LR-induced hepatic tissue inflammation, oxidative stress, and lipid metabolic disorders in darkbarbel catfish.

## 1. Introduction

Due to human activity and global warming, cyanobacterial blooms have emerged as a major global environmental threat [1,2]. Cyanobacteria proliferate in eutrophic waters and produce harmful cyanotoxins that can accumulate in aquatic ecosystems. Microcystins (MCs) are the largest group of cyanotoxins and are commonly found in rivers, lakes, streams, and ponds [3]. MCs are difficult to degrade in water and cause persistent harm to the aquatic ecosystem [4]. Among MCs, microcystin-leucine arginine (MC-LR) is recognized as the most toxic variant [5,6,7]. Extensive studies have demonstrated that MC-LR exhibits acute toxicity, including hepatotoxicity, neurotoxicity, genotoxicity, and reproductive toxicity [8,9,10], targeting multiple organs to induce damage. The World Health Organization (WHO) has recommended that the concentration of MC-LR in drinking water should not exceed 1 μg/L.

The liver is the primary target organ for MC-LR [11,12]. It is transported to the liver via the organic anion transporting polypeptide systems and bile acid transports [13,14,15]. More than 50% of ingested MCs accumulate in the liver [16]. MC-LR inhibits the activity of cellular protein phosphatases 1/2A (PP1/PP2A), activating Akt and p38/ERK/JNK pathways, resulting in hepatocyte proliferation [17,18,19]. It triggers hepatic inflammation by activating signaling pathways such as Gankyrin, PI3K/AKT, HIF-1α, RAC1/JNK, and NEK2, ultimately leading to carcinogenesis [20]. MC-LR also elevates reactive oxygen species (ROS) levels, which induce mitochondrial damage, endoplasmic reticulum stress, and apoptosis [21].

Following recent advancements in metabolomics, the perturbation of hepatic metabolism in response to MC-LR exposure is of great interest to physiologists. The precise quantification of endogenous metabolites sensitively reflects the liver’s stress response to MC-LR [22]. Recent studies demonstrate that glucose and lipid metabolism are disrupted by MC-LR exposure, leading to irreversible injury [23,24,25,26]. Following MC-LR treatment, alterations in serum metabolites, including triglycerides (TG), unsaturated fatty acids (UFAs), and very low-density lipoprotein (VLDL) levels, were observed, indicating that MCs severely disrupt hepatic lipid metabolism in mice [25]. MC-LR promotes the development of non-alcoholic steatohepatitis (NASH) by disrupting lipid metabolism [27]. The systematic study of metabolic responses not only elucidates the toxic mechanisms of MC-LR, but also provides critical biomarkers for the clinical diagnosis of diseases induced by MC-LR.

Although the hepatotoxicity of MC-LR has been extensively studied, most research focuses on murine models. Metabolic response studies in fish remain scarce. Compared to terrestrial animals, fish are disproportionately vulnerable to MC-LR [11,28]. Due to their high tolerance, fish exhibit pronounced bioaccumulation for MCs. When water bodies are contaminated, MC content can reach up to 874 μg/g dry weight in fish liver [29]. This bioaccumulation raises food safety concerns, which pose a significant threat to human health [30]. Consequently, investigating the toxicological mechanisms of MC-LR on fish is critically important for both aquatic ecosystem health and food safety.

Exposure to MCs in aquatic ecosystems is typically characterized by relatively low concentrations over prolonged periods [31,32,33]. However, subchronic exposure to environmentally relevant concentrations of MC-LR has received limited attention due to low mortality rates and statistically insignificant changes in target biomarkers. In reality, the detrimental effects of subchronic stress are often more persistent and insidious. An epidemiological study investigating long-term MC exposure in fishermen from Taihu Lake revealed that prolonged exposure may result in liver damage and increase the risk of developing non-alcoholic fatty liver disease (NAFLD) [34].

The darkbarbel catfish (*Tachysurus vachelli*) is a common scaleless fish species widely distributed in the river and lake ecosystems of China. Owing to its excellent taste and high nutritional value, it has emerged as an important aquaculture species favored by consumers. With increasing deterioration of water quality, cyanobacterial blooms frequently occur in fish ponds during summer, often persisting for approximately one month [35]. Consequently, the darkbarbel catfish is particularly susceptible to MC-LR stress. In this study, fish were exposed to environmentally relevant concentrations of MC-LR (0, 2, and 5 μg/L) for 28 days and sampled on days 14 (D14) and 28 (D28). This study aims to simulate natural conditions and investigate the effects of subchronic MC-LR exposure on hepatic histology, antioxidative status, and hepatic metabolism. Furthermore, untargeted metabolomic analysis was employed to provide novel insights into the metabolic responses to MC-LR exposure.

## 2. Results

### 2.1. Histopathological Examination

**Figure 1:**
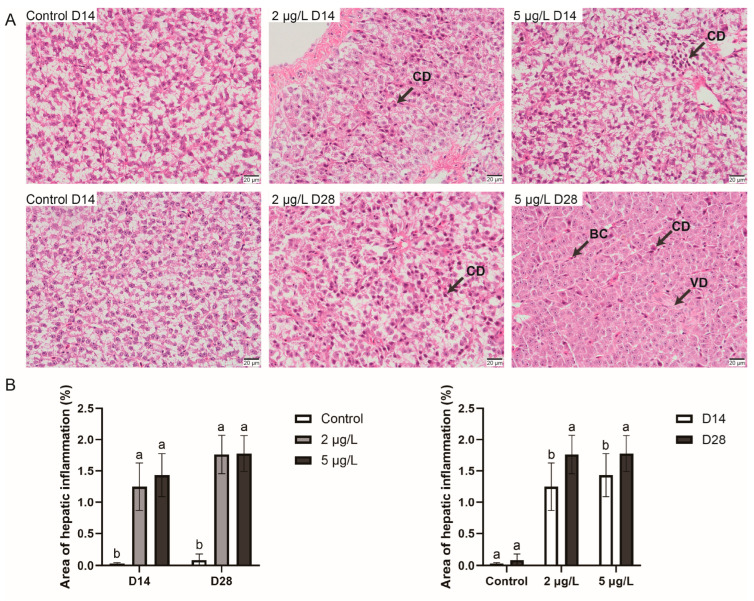
(**A**) Hepatic histopathological sections (400× magnification). CD, cellular degeneration; BC, blood cells; VD, vacuolar degeneration. (**B**) Hepatic inflammation area across exposure durations and concentrations. Data are mean ± SEM. (*n* =10). Different superscripts indicate significant differences (*p* < 0.05).

To assess the degree of tissue injury, pathological sections from different treatment groups across various experimental time points were compared. As shown in Figure 1A, liver sections revealed significant hepatic injury in the treatment groups. In contrast to the control groups, which exhibited intact hepatocytes with normal morphology and well-defined boundaries, both the 2 μg/L and 5 μg/L groups demonstrated hepatocellular degeneration characterized by indistinct cellular boundaries, indicative of inflammation induced by MC-LR exposure. No significant difference was observed in the area of hepatic inflammation between the different concentrations of MC-LR treatment groups. The severity of inflammation increased with longer exposure durations (Figure 1B). Notably, in the 5 μg/L group after 28 days of exposure, blood cells were observed intermingled among the hepatocytes, and vacuolization was evident in some hepatic cells.

### 2.2. Hepatic Biochemical Parameters

**Figure 2:**
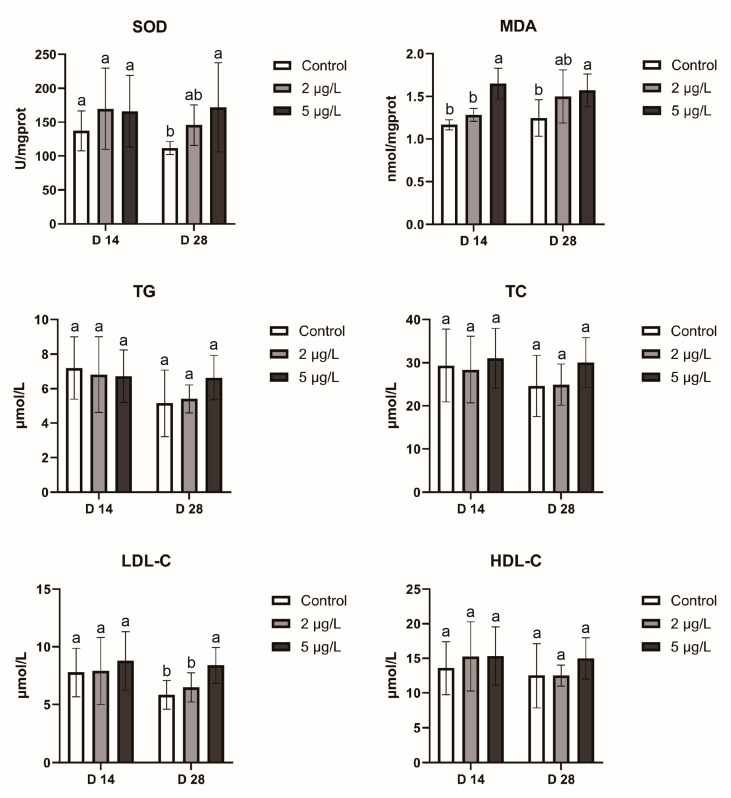
Hepatic enzymes activities and lipid profiles in darkbarbel catfish across treatments (*n* = 6). Data are shown as mean ± standard deviation (SD). Different superscripts indicate significant differences (*p* < 0.05).

Hepatic oxidative stress was assessed by measuring superoxide dismutase (SOD) activity and levels of malondialdehyde (MDA), as shown in Figure 2. Specifically, both hepatic SOD activity and MDA levels were significantly elevated (*p* < 0.05) following MC-LR exposure, except for SOD activity at D14 (*p* > 0.05). The levels of triglycerides (TG), total cholesterol (TC), low-density lipoprotein cholesterol (LDL-C), and high-density lipoprotein cholesterol (HDL-C) collectively reflect the status of hepatic lipid metabolism. Notably, LDL-C levels in the 5 μg/L group at D28 were significantly higher (*p* < 0.05) than those in both the control and 2 μg/L groups.

### 2.3. Differentially Abundant Metabolites Analysis

**Figure 3:**
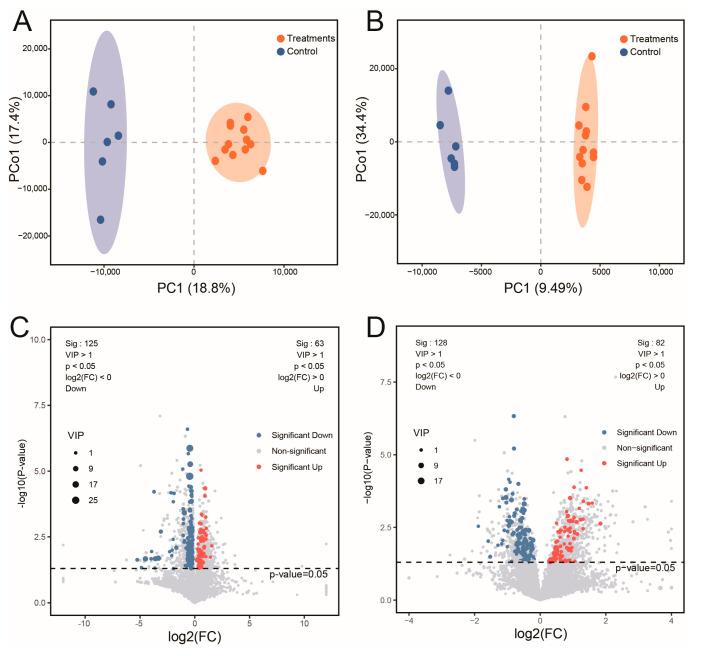
Analysis of hepatic differentially abundant metabolites (DAMs) between MC-LR and control groups. (**A**) OPLS-DA score plot (D14); (**B**) OPLS-DA score plot (D28); (**C**) Volcano plot (D14); (**D**) Volcano plot (D28).

A total of 5992 metabolites were identified in the liver. Orthogonal partial least-squares-discriminant analysis (OPLS-DA) demonstrated distinct variations in metabolite profiles across treatment groups. The principal component 1 (PC1) effectively segregated the treatment groups from the controls (Figure 3A,B). Volcano plots provided additional characterization of these variations (Figure 3C,D). After 14 days of exposure, 188 differentially abundant metabolites (DAMs) were identified (variable importance in projection, VIP > 1; *p* < 0.05), comprising 125 down-regulated and 63 up-regulated metabolites. Following 28 days of exposure, 210 DAMs were detected (128 down-regulated, 82 up-regulated).

**Figure 4:**
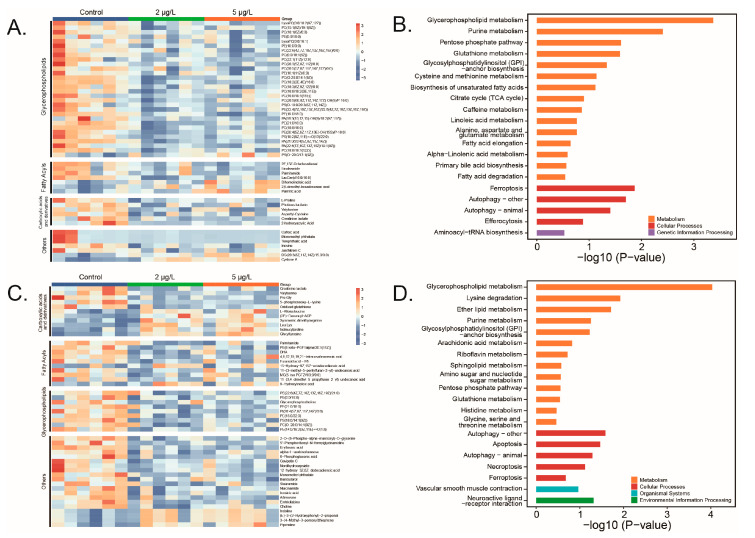
Top 50 differentially hepatic differentially abundant metabolites (DAMs). (**A**) Heatmap (D14); (**B**) pathway enrichment (D14); (**C**) heatmap (D28); (**D**) pathway enrichment (D28).

The top 50 DAMs ranked by VIP values were clustered to visualize variations in metabolite abundance across different samples (Figure 4A,C). At each time point, the majority of the top 50 DAMs were classified as lipid molecules (74% at D14 and 38% at D28), including glycerophospholipids (GPs) and fatty acyls. Following MC-LR exposure, the abundance of key GPs, such as phosphatidylcholine (PC), phosphatidylethanolamine (PE), phosphatidylserine (PS), and phosphatidic acid (PA), showed significant reductions.

### 2.4. KEGG Enrichment

**Figure 5:**
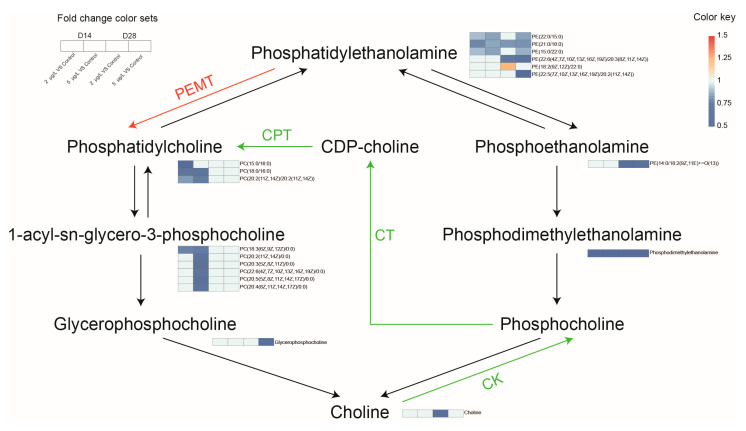
Alterations of key glycerophospholipids (GPs) in phosphatidylcholine (PC) synthesis pathways after MC-LR exposure. Fold changes of each metabolite shown in a heatmap (blue: downregulation; orange: upregulation). The red arrow represents the PEMT pathway. The green arrows represent the Kennedy pathway. PEMT, phosphatidylethanolamine N-methyltransferase; CK, choline kinase; CT, cytidylyltransferase; CPT, cholinephosphotransferase.

Pathway enrichment analysis using the KEGG ID of DAMs indicated that GP metabolism was the most significant altered metabolic pathway at both D14 (*p* < 0.01, q < 0.05) (Figure 4A) and D28 (*p* < 0.01, q < 0.01) (Figure 4D). Significant variations in the levels of seven metabolites, including PC, PE, 1-acyl-sn-glycero-3-phosphocholine (LysoPC), glycerophosphocholine (GPC), phosphoethanolamine (PEA), phosphodimethylethanolamine, and choline were detected (Figure 5). These metabolites are involved in PC synthesis within GP metabolism. Most of them decreased following MC-LR exposure.

### 2.5. Trend Analysis

**Figure 6:**
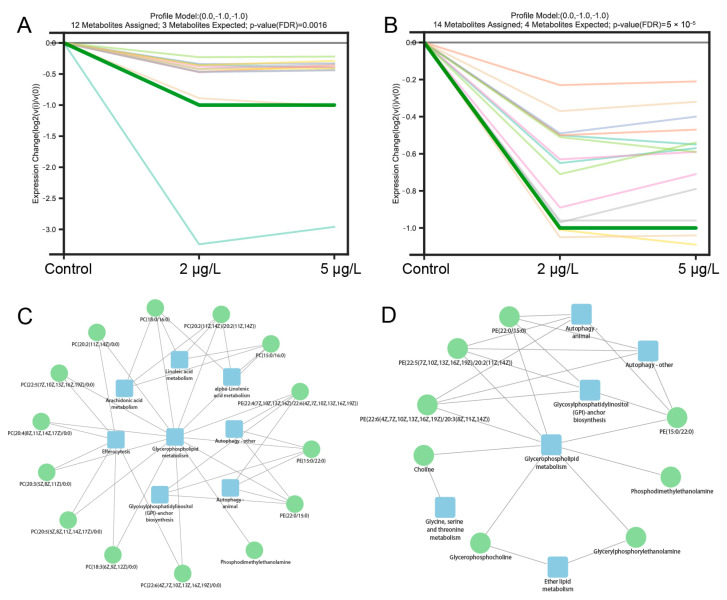
Trend analyses of metabolites and metabolic pathways. (**A**,**B**) Variation patterns of metabolites across MC-LR concentrations at D14 and D28, respectively, and different line colors represent different metabolites; (**C**,**D**) metabolite−pathway interaction networks at D14 and D28, respectively. Blue rectangles denote metabolic pathways; green circles indicate differentially abundant metabolites (DAMs).

We examined 16 different variation-trend patterns of DAMs under varying concentrations of MC-LR. Metabolites were significantly assigned to the profile model (0.0, −1.0, and −1.0) in both the D14 and D28 phases (Figure 6A,B). No significant associations were observed for the remaining 15 patterns. The results of the metabolite−pathway interaction network analysis indicated that GP metabolism was associated with the highest number of metabolites at D14 (Figure 6C). These 14 metabolites were also linked to seven other metabolic pathways, e.g., fatty acid metabolism, autophagy, efferocytosis, and GPI-anchor biosynthesis. However, after 28 days of exposure to MC-LR, GP metabolism was only associated with 8 metabolites and 5 metabolic pathways (Figure 6D).

## 3. Discussion

While the hepatotoxicity of MC-LR is well-documented [11,20], this study provides novel insight into the impacts of exposure to environmentally relevant concentrations, which is particularly pertinent for elucidating the toxicological effects on fish. A damage model was established under MC-LR stress, and no mortality was observed throughout the experiment. Histopathological analyses revealed that the experimental fish were highly susceptible to low-concentration MC-LR stress. Hepatic damage was evident in all treatment groups, with cellular degeneration, hyperemia, and inflammation being the primary manifestations of liver injury. These findings were consistent with a previous study on dietary cyanobacteria exposure in yellow catfish [33].

Oxidative stress is widely acknowledged as a critical pathophysiological indicator [36,37]. The equilibrium between the promotion and suppression of oxidative stress plays a pivotal role in maintaining organismal health [38,39]. Extensive studies have demonstrated that MC-LR exposure can contribute to mitochondrial injury and significantly elevate reactive oxygen species (ROS) levels [40,41,42]. Increased ROS activity triggers lipid peroxidation cascades, forming reactive lipid peroxides, such as MDA [43,44]. To counteract these effects, organisms activate antioxidant systems, which induce detoxifying enzymes like SOD [45]. In *Cyprinus carpio*, MDA levels exhibited a significant increase 2–6 days after exposure to 10 μg/L of MC-LR, confirming MDA’s sensitivity to MC-LR [46]. In this study, a significant increase in MDA levels (*p* < 0.05) was observed in the 5 μg/L treatment groups at both D14 and D28, indicating that exposure to low concentrations of MC-LR (5 μg/L) can also induce oxidative stress. At D14, no statistically significant difference in SOD activity was observed between the control and MC-LR treatment groups, suggesting that SOD exhibits a delayed response to MC-LR compared to MDA. These findings suggest that using SOD activity as a biomarker to assess oxidative stress status in darkbarbel catfish may lead to diagnostic inaccuracies under MC-LR exposure conditions. A compensatory upregulation of SOD activity was detected at D28, with the highest increase (54%; *p* < 0.01) occurring in the 5 μg/L group. Despite this compensatory upregulation of SOD activity, subchronic exposure to environmentally relevant concentrations of MC-LR induces persistent lipid peroxidation.

MC-LR exposure is well-documented to disrupt hepatic lipid metabolism across experimental models, including mice [25,27], fish [14,47], and frogs [48]. In a 28 day gavage experiment, mice treated with MC-LR exhibited significantly elevated hepatic TG and HDL-C levels alongside reduced TC and LDL-C levels relative to controls [25]. Conversely, fasted frogs exposed to MC-LR showed increased hepatic TC but decreased hepatic TG [48]. In the present study, most lipid profiles remained unchanged under low-dose exposure. Similar patterns occurred in Nile tilapia, with 60 day MC-LR exposure producing no significant lipid profile differences in low-concentration groups [47]. Thus, low-concentration MC-LR exposure likely explains these non-significant lipid profile alterations. Nevertheless, the marked LDL-C elevation at D28 suggests potential hepatic lipid metabolism disruption.

The liver functions as a central organ for lipid metabolism. TC and TG are synthesized hepatically, where they combine with phospholipids to form lipoprotein complexes [49,50]. These complexes are transported via high-density lipoprotein (HDL) and low-density lipoprotein (LDL) to peripheral tissues, fulfilling systemic metabolic demands including steroidogenesis and membrane biosynthesis [51]. Prior research established significant associations between the GP metabolic pathway and hepatic lipid disorders, highlighting the critical role of GP metabolism in disease pathogenesis [24,27]. PC, the predominant GP in eukaryotes, is essential for lipoprotein assembly and secretion [52,53]. Impaired PC biosynthesis inhibits lipoprotein formation, disrupting lipid transport and potentially triggering non-alcoholic fatty liver disease (NAFLD) [27,54]. PC synthesis occurs through two distinct pathways: the Kennedy pathway and the phosphatidylethanolamine N-methyltransferase (PEMT) pathway [55,56,57]. Under choline-sufficient conditions, PC is primarily synthesized via the Kennedy pathway using choline as a substrate. During choline deprivation, PE serves as the PC precursor through the PEMT pathway in the endoplasmic reticulum [58]. In this study, PC and its intermediates (choline, PE, PEA, and GPC) decreased significantly following MC-LR exposure. Reduced PE availability hindered the PEMT pathway, while diminished choline impaired the Kennedy pathway. This disrupted PC biosynthesis potentially inhibits hepatic lipoprotein secretion, thereby promoting inflammation.

Different exposure times and concentration gradients were established to investigate MC-LR-induced damage progression. Contrary to expectations, low-dose MC-LR exposure did not significantly alter hepatic inflammation progression. No significant up- or down-regulation trends were observed in DAMs shared across concentration gradients. For instance, on day 14, 12 DAMs common to T1 and T2 groups conformed to the profile model (0.0, −1.0, −1.0), indicating that metabolite levels plateaued despite increasing MC-LR concentrations. A similar pattern occurred on day 28. The subsequent metabolite−pathway interaction network analysis highlighted the critical role of GP metabolism in MC-LR stress response. As the primary targeted pathway shifted from GP metabolism to others between D14 and D28, liver injury intensified with prolonged MC-LR exposure. These findings suggest that MC-LR-induced damage initiates through GP metabolism disruption and intensifies via cross-pathway interactions. Aberrant GP metabolism perturbed fatty acid metabolism and cellular processes (e.g., autophagy and efferocytosis), amplifying hepatic inflammation.

The identification of biomarkers for hepatic poisoning diagnosis represents a critical future research direction. This study demonstrated the sensitivity of endogenous metabolites to MC-LR stress. Even at low exposure concentrations, GPs still exhibited marked alterations, suggesting their utility as indicators of hepatic toxic stress response. Notably, GP alterations vary across species and toxic conditions. Zebrafish co-exposed to polystyrene microplastics and cypermethrin showed increased hepatic GPs [59], while MC-LR exposure elevated hepatopancreatic GP levels in Pacific white shrimp within 72 h [60]. In our study, GP alterations also varied with exposure duration. These observations highlight that individual metabolite changes alone are insufficient for assessing hepatic damage severity. Collectively, the findings underscore the complexity of toxin-induced GP metabolic disruption and liver injury, which depends on the species, toxin type, and target organ. Additionally, factors like co-occurring stressors and sex-specific effects remain underexplored in this study.

## 4. Materials and Methods

### 4.1. Ethics Statement and Sample Collection

The experimental protocol was approved by the Experimental Animal Welfare and Ethical Committee of Anhui Academy of Agricultural Sciences (approval no. AAAS 2023-33). A total of 270 one-year-old darkbarbel catfish (*T. vachelli*) were obtained from Huarun Technology Aquaculture Co., Ltd., Lu’an, China, and randomly allocated into nine tanks (70 L water volume per tank). The average body weight was 35.19 ± 8.04 g, with no significant inter-tank differences observed.

### 4.2. Exposure Experiments and Sampling

**Figure 7:**
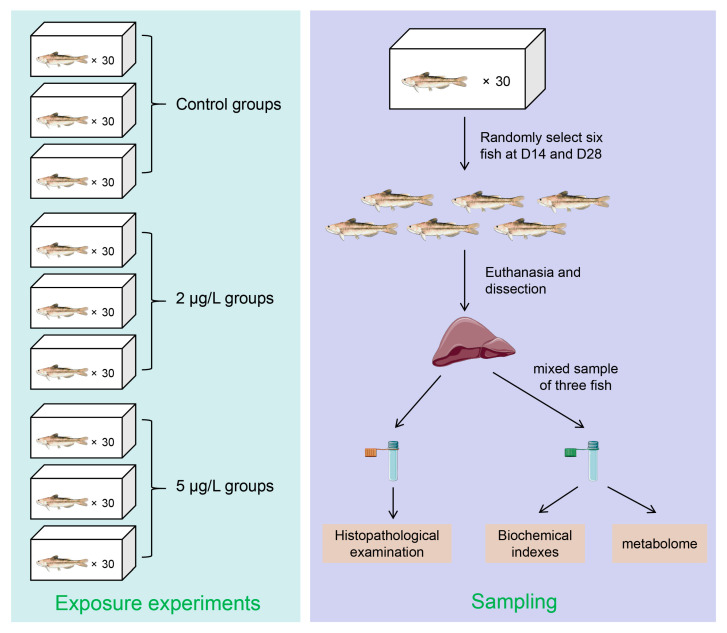
Experimental design. The liver and tube icons by Servier (https://smart.servier.com/, accessed on 28 May 2025) are licensed under CC-BY 3.0 Unported (https://creativecommons.org/licenses/by/3.0/, accessed on 28 May 2025).

After a 7-day acclimation period, fish were randomly allocated into three treatment groups (0, 2, and 5 μg/L MC-LR), each with three replicate tanks (Figure 7). Concentrations were determined based on environmentally relevant levels measured in fish ponds [32,35]. MC-LR (purity ≥ 98%; Naturewill Biotechnology Co., Ltd., Chengdu, China) was diluted to target concentrations using dechlorinated tap water. Fish were maintained under these conditions for 28 days with daily feeding at 2% of the body weight. During the experiment, 80% of the water in each tank was replaced every two days to maintain MC-LR concentrations. On D14 and D28, six fish per tank were randomly sampled. The selected fish were euthanized by rapid freezing. Liver sections were fixed in 4% paraformaldehyde for histopathological analysis. The remaining liver tissues were flash-frozen in liquid nitrogen for subsequent metabolomic analysis.

### 4.3. Hematoxylin-Eosin Staining

Fixed liver tissues were dehydrated using ethanol, embedded in paraffin wax, and sectioned at 4 μm thickness. The sections were stained with hematoxylin−eosin (H&E; Servicebio, Wuhan, China) and examined under an Olympus BX43 light microscope (Tokyo, Japan). The area of hepatic inflammation was measured using ImageJ version 1.54.

### 4.4. Biochemical Indexes Analysis

Biochemical parameters were measured on the day of sample collection. Liver tissues were homogenized in 1× PBS (pH 7.4) to prepare 10% (*w*/*v*) tissue homogenates. Biochemical parameters, including MDA, SOD, LDL-C, HDL-C, TC, and TG, were quantified using enzyme-linked immunosorbent assay (ELISA) kits (Bioroyee Biotechnology Co., Ltd., Beijing, China).

### 4.5. Liver Metabolomic Analysis

Liver tissues from three fish per tank were randomly pooled as a single biological replicate. The tissues were precooled in 80% methanol at −40 °C for 2 min, followed by ultrasonic extraction in ice water. The solution was centrifuged at 13,000 rpm for 20 min. After filtration, 150 μL of supernatant was injected into the LC-MS/MS system for analysis.

Untargeted metabolomic analysis was performed using an Acquity UPLC I-Class Plus (Waters Corporation, Milford, MA, USA) coupled with a Q-Exactive mass spectrometer (Thermo Fisher Scientific, Waltham, MA, USA). Metabolites were annotated by referencing the Human Metabolome Database (HMDB), Lipidmaps (version 2.3), and Metlin database. OPLS-DA was employed to identify the DAMs between groups. VIP values derived from the OPLS-DA model were utilized to rank the contribution of each variable to group discrimination. The significance of DAMs was assessed using a two-tailed Student’s *t*-test. DAMs were screened based on the criteria VIP > 1 and *p* < 0.05. Additionally, trend analysis of metabolite changes was performed using short time-series expression miner (STEM) software version 0.1.6 (available at https://cloud.oebiotech.cn/task/detail/stem/).

### 4.6. Statistical Analysis and Graph Visualization

Data were tested for normality using the Shapiro−Wilk test. One-way analysis of variance (ANOVA), followed by a least significant difference (LSD) test was performed to assess significant differences. Graphical visualization was achieved using the online analytical platform available at https://cloud.oebiotech.com/#/bio/tools.

## Data Availability

The original contributions presented in this study are included in the article. Further inquiries can be directed to the corresponding authors.
